# High dietary Ca and microbial phytase reduce the expression of Ca transporters while enhancing claudins involved in paracellular Ca absorption in the porcine jejunum and colon

**DOI:** 10.1017/S0007114522002239

**Published:** 2023-04-14

**Authors:** Yixin Hu, Jurgen van Baal, Wouter H. Hendriks, Jan-Willem Resink, Annette Liesegang, Marinus M. van Krimpen, Paul Bikker

**Affiliations:** 1 Wageningen University and Research, Wageningen Livestock Research, Wageningen, 6700, the Netherlands; 2 Wageningen University and Research, Animal Nutrition Group, Wageningen, the Netherlands; 3 Trouw Nutrition Research and Development, Boxmeer, the Netherlands; 4 University of Zurich, Institute of Animal Nutrition, Vetsuisse Faculty, Zürich, Switzerland

**Keywords:** Jejunum and colon, Metabolism, Calcium and phosphorus transporters, Claudins, Pig

## Abstract

Expression levels of genes (RT-qPCR) related to Ca and P homeostasis (transporters and claudins (CLDN)) were determined in porcine jejunal and colonic mucosa. Forty growing pigs (BW 30·4 (sem 1·3) kg) received a low and high Ca content (2·0 and 9·6 g/kg, respectively) diet with or without microbial phytase (500 FTU/kg) for 21 d. Dietary Ca intake enhanced serum Ca and alkaline phosphatase concentration and reduced P, 1,25(OH)_2_D_3_, and parathyroid hormone concentration. Jejunal transient receptor potential vanilloid 5 (TRPV5) mRNA expression was decreased (32%) with phytase inclusion only, while colonic TRPV5 mRNA was reduced by dietary Ca (34%) and phytase (44%). Both jejunal and colonic TRPV6 mRNA expression was reduced (30%) with microbial phytase. Calbindin-D9k mRNA expression was lower in colonic but not jejunal mucosa with high dietary Ca (59%) and microbial phytase (37%). None of the mRNAs encoding the Na–P cotransporters (NaPi-IIc, PiT-1, PiT-2) were affected. Jejunal, but not colonic expression of the phosphate transporter XPR1, was slightly downregulated with dietary Ca. Dietary Ca downregulated colonic CLDN-4 (20%) and CLDN-10 (40%) expression while CLDN-7 was reduced by phytase inclusion in pigs fed low dietary Ca. Expression of colonic CLDN-12 tended to be increased by phytase. In jejunal mucosa, dietary Ca increased CLDN-2 expression (48%) and decreased CLDN-10 (49%) expression, while phytase slightly upregulated CLDN-12 expression. In conclusion, compared with a Ca-deficient phytase-free diet, high dietary Ca and phytase intake in pigs downregulate jejunal and colonic genes related to transcellular Ca absorption and upregulate Ca pore-forming claudins.

The two essential macrominerals Ca and P regulate a wide range of biochemical and cell signalling processes and are the principal constituents of bone in terrestrial organisms^(1)^. Intestinal absorption of these minerals from the diet plays an important role in the regulation of whole body Ca and P homeostasis. Ionised Ca is absorbed predominantly in the small intestine and partly in the colon, accounting for approximately 80 and 20 % of the total tract Ca absorption in pigs, respectively^(2)^. The uptake of these minerals occurs either transcellular or paracellular with the former involving Ca transport proteins calbindin-D9k/-D28k (CaBP-D9k/-D28k), sodium (Na)–Ca exchanger (NCX1) in concert with plasma membrane Ca-ATPase 1 (PMCA1)^(3)^. The components responsible for transcellular P uptake are less clear. Experimental data showed that the apical step is mediated mainly by members of the Na-dependent inorganic phosphate cotransporter type II (NaPi-II) family^(4)^. Based on studies in rats and mice, phosphate inorganic transporter 1 (PiT-1) and 2 (PiT-2) also appear to play a role in active intestinal P absorption^(5)^. In addition, it has been proposed that the basolateral step could be mediated by xenotropic and polytropic retrovirus receptor 1 (XPR1/SLC53A1)^(6)^. While transcellular transport is an active, ATP consuming process, paracellular transport is dependent on passive diffusion. Diffusion of Ca and P occurs through a complex of tight junction proteins, that enclose the apical and lateral membranes of enterocytes. In this context, claudins (CLDN) are the most important transmembrane components of the tight junction complex, comprising barrier (i.e. tightening) and permeability-mediating members^(7)^. The latter group includes cation-selective CLDN-2 and CLDN-12, which build pores highly permeable for Ca^(8)^. In addition, CLDN-10b^(9)^ and CLDN-15^(10)^ can modulate small intestinal Na permeability. The anion-selective CLDN members, on the other hand, are largely unknown as inconsistent results have been reported. Depending on cell type, CLDN-4^(11)^ and CLDN-7^(12)^ have been shown to form pores permeable for chloride (Cl) but a barrier to Na. Another tight junction protein is zonula occludens protein 1 (ZO-1/TJP1), which ensures a stable connection to the cytoskeleton of the epithelial cell^(13)^. Understanding the molecular mechanisms underlying the modulation of intestinal Ca and P transporters through dietary intervention is an important topic in livestock in order to further improve performance and health status of animals, assure efficient utilisation of Ca and P, alleviate legal pressure on P output and minimise environmental pollution.

Recently, we reported that growing pigs fed microbial phytase supplemented diets displayed improved inositol phosphate (IP) degradation and enhanced apparent absorption of Ca and P in the small intestine^(2)^. Strikingly, the phytase-free diets resulted in the increased passage of substantial amounts of P towards the large intestine, where it was absorbed three times more compared with phytase-supplemented diets. Furthermore, apparent absorption of Ca in the colon was approximately 2-fold greater with phytase-free diets. Similar findings have been reported by Gonzalez-Vega *et al.* ^(14)^, who investigated Ca and P absorption in various gut segments in pigs receiving semi-purified diets. They demonstrated that apparent P absorption in the colon was substantial in the IP supplemented but not in IP-free diets. These observations imply that colonic Ca and P absorption is dependent on luminal microbial phytase activity and its IP content. It is conceivable that phytase supplementation enhanced IP degradation in the distal region of the small intestine in our previous study^(2)^. Consequently, less IP would be available for the microbiota residing in the colon to release Ca and P ions and inositol from the IP complex for colonic absorption. In addition, mucosal phosphatases (e.g. multiple inositol polyphosphate phosphatase 1 (MINPP1) and intestinal alkaline phosphatase (IAP)) might also be reduced because less substrate was available in the colon. However, molecular mechanisms underlying the regulation of colonic Ca and P absorption remain largely unknown. Because of the high viscosity of digesta and the lower permeability of the colon to defend against pathogens and fermentation toxins (e.g. phenol, cresol), colonic Ca and P absorption is more likely to be mediated via the more controllable, active transcellular pathway. The aim of the research described in this paper was to assess the effect of dietary Ca level and microbial phytase supplementation on the expression of genes related to Ca, P and inositol absorption as well as mucosal phosphatases in the jejunum and colon. We hypothesised that the expression level of genes involved in transcellular and paracellular transport in the colon of pigs would be greater when feeding diets not supplemented with phytase to favour the absorption of these two minerals.

## Materials and methods

The work described here is a follow-up of our recent *in vivo* pig study in which the interactive effects of dietary Ca level and microbial phytase supplementation on Ca and P absorption and solubility along the intestine in growing pigs were investigated^(2)^. From that study, comprising two dietary levels of microbial phytase (0 *v*. 500 FTU/kg) and three levels of Ca (2·0, 5·8 and 9·6 g/kg) in a 2 × 3 factorial arrangement, results of the low (2·0 g/kg) and high (9·6 g/kg) Ca level with (500 FTU/kg) or without dietary phytase groups were selected for the gene expression analysis, while results of all six treatments were selected for serum analysis in the present study. This study was approved by the ethical committee of Wageningen University & Research (2016.D-0065.006) and conducted in the facilities of the Swine Research Centre of Trouw Nutrition (Sint Anthonis, the Netherlands). All procedures were in agreement with the Dutch laws on animal experiments.

### Experimental design and diets

Details regarding the experimental design, feed composition, animal husbandry, feeding regime, as well as sample collection, are described in our previous study^(2)^. Briefly, forty young growing pigs (Hypor Libra×Maxter), weighing 30·4 (sem 1·3) kg, were randomly allotted to two dietary levels of microbial phytase (0 *v*. 500 FTU/kg) and three levels of Ca (2·0, 5·8 and 9·6 g/kg) in a 2 × 3 factorial arrangement. Dietary Ca content met was below or greater than the minimal Ca requirement for growing pigs for the applied medium, low and high Ca levels. From that study, results of the low (2·0 g/kg) and high (9·6 g/kg) Ca level with (500 FTU/kg) or without dietary phytase groups were selected for gene expression analysis in the present study. Gene expression of pigs fed the middle Ca diet was expected to be in line with the low and high Ca diets, as previous study^(15)^ have shown increasing dietary Ca content from 2·0 to 12·1 g/kg linearly reduced mRNA expression of Ca-related transporters in the duodenum of pigs. Hence both Ca under- and oversupply can regulate the expression of Ca transporters and CLDN. No limestone was added to the basal diet, and monosodium phosphate was used to realise the intended marginal digestible P content (1·7 g/kg). As reported in our previous study^(2)^, 500 FTU microbial phytase increased apparent Ca absorption by 0·7 and 0·9 g/kg for the low and high Ca diets, respectively. Furthermore, microbial phytase also enhanced apparent P absorption by 1·4 and 1·1 g/kg for the low and high Ca diets, respectively^(2)^. The experiment was replicated over time with two runs of five replicate pigs/treatment/run. Pigs were blocked by their initial body weight (BW) with pigs within a block randomly allocated to one of the six experimental diets for 21 d. The analysed Ca, P and IP content as well as phytase activity in the diets were in good agreement with the targeted values for all treatment groups^(2)^.

### Sample collection and treatment selection

At the end of the experiment, pigs (BW 44·6 (sem 2·0) kg) were sedated with Zoletil^®^ 100 (0·06 ml/kg BW) and then killed via jugular vein injection of Euthasol^®^ 100 (24 mg/kg BW). Blood was collected from the carotid artery before exsanguination and centrifuged at 3000 × g for 10 min at 4 °C to harvest serum. Subsequently, the gastrointestinal tract (GIT) was dissected out and emptied by gentle squeezing before the mucosa was cleansed with water and scraped from the middle of jejunum and colon, frozen in liquid nitrogen and stored at −80 °C until further analysis.

### Serum analysis

Serum Ca and P were analysed using a Cobas 8000 modular analyser with C701 Photometric measuring unit (Roche Diagnostics Limited, Rotkreuz, Switzerland). Commercially available test kits were used to analyse serum parathyroid hormone (PTH, Immunotopics), 25-hydroxycholecalciferol (25(OH)D_3_) and 1,25-dihydroxycholecalciferol (1,25(OH)_2_D_3_, Immunodiagnostic Systems GmbH) and alkaline phosphatase (ALP, Diatools AG).

### Real-time quantitative PCR analysis

The RNA isolation and gene expression determination were conducted following the standard protocol in our laboratory. Briefly, deep-frozen jejunum and colon mucosa were ground in liquid N_2_ and used for total RNA isolation using TRIzol (ThermoFisher Scientific). The RNA was subjected to on-column DNAse treatment to remove possible genomic DNA contamination with the Nucleospin II kit (Macherey Nagel). Quantity and quality of RNA were determined with the NanoDrop 1000 Spectrophotometer (ThermoFisher Scientific) and 2100 Bioanalyzer and RNA 6000 Nano LabChip kit (Agilent Technologies), respectively. The RNA integrity number values ranged from 9·7 to 10. A total of 500 ng RNA was reverse transcribed with Superscript III kit (ThermoFisher Scientific) and mRNA levels were assessed by real-time quantitative PCR (RT-qPCR) amplification on a QuantStudio 5 Real-Time PCR System (ThermoFisher Scientific) using the SensiFAST™ SYBR^®^ low-ROX Kit (Bioline) under the following conditions: 95 °C for 15 s and 60 °C for 30 s for 40 cycles. The used primer sequences were designed with Primer Express Software (Life Technologies, Bleiswijk, the Netherlands), and where possible recommended primer sets that span an intron were selected and presented in Table 1. Absolute mRNA levels of genes of interest were normalised to the endogenous expression level of importin 8 (IPO8), since Normfinder^(16)^ pointed out that IPO8 was the most stable gene compared with that of eukaryotic elongation factor 2 (EEF2) and beta actin (ACTB).

**Table 1.** Primers used for RT-qPCR determination

Genes	Accession number	Forward (5′–3′)	Reverse (5′–3′)
Ca transporters			
CaSR	XM_021068447	TTGCTCATGCCCTGCAAGAT	AAGTGCCGTAGGTGCTTCAG
CaBP-D9k	NM_214140	TATGCAGCCAAAGAAGGGGAT	CTAGGGTTCTCGGACCTTTCAG
CaBP-D28k	NM_001130226	GGCTTCATTTCGACGCTGAC	TCGGGTGATAACTCCAATCCAG
NCX1	XM_021088333	GTTTGTGGCGCTTGGAACTT	TGCCCGTGACGTTACCTATG
TRPM7	XM_013993003	AGCACCATCTTGGACTCTTGC	CCCAGGACCACAGATTTCACG
TRPV5	XM_021078896	GCTGCAACAGAAGAGGATTCG	CCGAAAGTCACAGGTTCGGT
TRPV6	XM_021078898	AGCAGAAGCGGATCTGGG	GGGCTCCTTTCTGGTGGACA
P transporters			
NaPi-IIa	XM_021082353	GACAGGACTGACTTCCGGC	CACGGATGTTGAAGGAGGCT
NaPi-IIb	NM_001256772	CTCTGTAGCTGCCGGGTCCTAA	GGTCAGAGTCGACGAGAACAC
NaPi-IIc	XM_021081190	GCATCCTGTTGTGGTACGTG	AGAAGCCGAGCAACAGGTAG
PiT-1	XM_021087023	GATGTTTGGTTCTGCTGTGTG	AGACCACTTGACACCCTCCTG
PiT-2	XM_021078067	TCACGAACCAAGCCACGAA	GATCTCACCCCCATCACACC
XPR1	XM_003130352	GGTGGCCTCTTGCCAAATGA	GCACTGGATAAAGCGAAGCC
Tight junctions			
CLDN-2	NM_001161638	CTGCCCACTGCAAGGAAATC	ACTCACTCTTGGCTTTGGGT
CLDN-4	XM_021085910	ATGGGTGCCTCGCTCTACAT	GAGTAGGGCTTGTCGGTACG
CLDN-7	XM_021066034	GCAGGTCTTTGTGCGTTGAT	AAGATGGCAGGGCCAAACTC
CLDN-10	XM_021065095	GACCGGGTGTTCCCTGTATG	GGCTCCTGCCCATCCAATAA
CLDN-12	NM_001160079	ATGACGTCCGTTCTGCTCTT	TACGTATGCATGCTGGGAGG
CLDN-16	NM_001161644	TTTTTGGCTGGTGCTGTCCT	CATACGACTTGGCCATCGAAAC
ZO-1	XM_021098891	GTTGGACAACCAGACGTGGA	ACTAACTTCATGCTGGGCCG
Other proteins			
CYP24	NM_214075	GGGCGGAGGATTTGAGGAAT	ATCAACACGGTTCCTTTGGGT
IAP	XM_003133725	CTAAAGGGGCAGATGAATGG	CACCTGTCTGTCCACGTTGT
MINPP1	XM_001927672	AGAAGCAAAGTTCTCAGCCAGTT	GGGGCTCCTTGTCTTTGAAGTA
SMIT	XM_005657149	CAAGGGGCCTTCTATGGTGG	CTGGCCTCTCATCAGGTTGG
VDR	NM_001097414	CTCTCCAGACACGATGGAGC	TTGGCAAAGCCGATGACCTT
Reference genes			
ACTB	XM_021086047	CGTGAGAAGATGACCCAGATCA	TCTCCGGAGTCCATCACGAT
EEF2	XM_003354002	AGTCCACTCTGACGGACTCA	AGAGGGAAATGGCCGTTGAT
IPO8	XM_003126400	TGCCATGGTATTTCTCCTCAAA	GCAGAAGAGGCATCATGTCTGT

ACTB, beta actin; CaBP-D9k, calbindin-D9k; CaBP-D28k, calbindin-D28k; CaSR, Ca sensing receptor; CYP24, vitamin D_3_ 24-hydroxylase; EEF2, eukaryotic elongation factor 2; IAP, intestinal alkaline phosphatase; IPO8, importin 8; MINPP1, multiple inositol polyphosphate phosphatase 1; NaPi-IIa (SLC34A1), sodium-dependent phosphate transporter type IIa; NaPi-IIb (SLC34A2), sodium-dependent phosphate transporter type IIb; NaPi-IIc (SLC34A3), sodium-dependent phosphate transporter type IIc; NCX1(SLC8A1), sodium-Ca exchanger; PiT-1 (SLC20A1), inorganic phosphate transporter 1; PiT-2 (SLC20A2), inorganic phosphate transporter 2; SMIT (SLC5A3), sodium/myo-inositol cotransporter; TRPM7, transient receptor potential cation channel subfamily M member 7; TRPV5, transient receptor potential cation channel subfamily V member 5; TRPV6, transient receptor potential cation channel subfamily V member 6; VDR, vitamin D_3_ receptor; XPR1 (SLC53A1), xenotropic and polytropic retrovirus receptor 1; ZO-1, zonula occludens-1.

### Data analysis

Pig was the experimental unit for all analysis. Serum and gene expression data were analysed by two-way ANOVA using the MIXED procedure of SAS (version 9.4, SAS Institute) with dietary phytase, Ca level and their interaction as fixed effects and batch and block as random effects. Distribution and homogeneity of variance of the Studentised residuals were visually checked via graphics plotted by the ODS GRAPHICS procedure. The LSMEANS procedure with a PDIFF option was used to separate means. Probability was considered significant at *P* ≤ 0·05 and a trend at 0·05 < *P* ≤ 0·1.

## Results

### Serum calcium, phosphorous, alkaline phosphatase, parathyroid hormone and vitamin D_3_

As expected, incremental dietary Ca content gradually increased the serum Ca content of the pigs (Table 2). This increase was dampened with the inclusion of microbial phytase (*P*_*interaction*_ < 0·001). Moreover, incremental dietary Ca reduced serum P content, with a greater effect in diets without phytase supplementation (*P*_*interaction*_ < 0·001). Serum ALP concentration increased with increasing dietary Ca (*P* < 0·001) but reduced with phytase supplementation (*P* = 0·030). Incremental dietary Ca level drastically decreased (*P* < 0·001) serum PTH concentration, and this inhibitory effect was significantly impeded with phytase inclusion in the medium Ca group (73 *v.* 5 pg/ml, *P*_*interaction*_ = 0·011). The serum concentration of the active form of vitamin D_3_, 1,25(OH)_2_D_3_ was, however, reduced by incremental dietary Ca level but only in combination with phytase supplementation (*P*_*interaction*_ = 0·010).

**Table 2.** Least squares means of various serum components as affected by dietary Ca level and microbial phytase supplementation in growing pigs*,†

Item	Dietary treatment							*P*-value		
Phytase, FTU/kg	0			500			Pooled	Ca	Phytase	Ca × Phytase
Ca, g/kg	2·0	5·8	9·6	2·0	5·8	9·6	sem			
Ca, mM	2·38^c,d^	2·78^b^	3·38^a^	2·35^d^	2·48^c^	2·82^b^	0·072	<0·001	<0·001	<0·001
P, mM	2·90^b^	2·19^d^	1·85^e^	3·23^a^	3·09^ab^	2·63^c^	0·098	<0·001	<0·001	<0·001
ALP, U/l	100	113	141	98	99	116	10·0	<0·001	0·030	0·280
PTH, pg/ml	118^a^	5^c^	1^c^	127^a^	73^b^	4^c^	15·9	<0·001	0·006	0·011
25(OH)D_3_, nM	70	86	89	84	102	110	6·4	<0·001	<0·001	0·710
1,25(OH)_2_D_3_, pM	1048^a^	1007^a^	1003^a^	1013^a^	873^b^	860^b^	26·3	<0·001	<0·001	0·010

ALP, alkaline phosphatase; PTH, parathyroid hormone; 25(OH)D_3_, 25-hydroxycalciferol; 1,25(OH)_2_D_3_, 1,25-dihydroxycalciferol.

* Dietary phosphorous content was fixed at 4·7 g/kg diet.

† Data are presented as treatment means, ten replicate pens per treatment (*n* 10).

^a–e^ Values lacking a common superscript within a row differ (*P* ≤ 0·05).

### Colonic expression levels

The effects of dietary Ca and microbial phytase supplementation on the gene expression levels of Ca and P transporters and tight junction proteins in the colonic and jejunal mucosa of the pigs are shown in Tables 3 and 4. Expression of colonic transient receptor potential vanilloid 5 (TRPV5) mRNA was reduced by 34 and 44% with high Ca intake and phytase inclusion (*P* = 0·030 and 0·004, respectively; Table 3). Similarly, high dietary Ca intake reduced TRPV6 and CaBP-D9k mRNA expression by approximately 55% (*P* < 0·001), and phytase reduced their expression to a less extent (approximately 35% reduction, *P* < 0·002). Neither the expression of NCX1 nor that of the none-selective cation channel, transient receptor potential cation channel subfamily M member 7 (TRPM7), were affected by the dietary treatments. Colonic expression of CaBP-D28k and the extracellular Ca sensing receptor (CaSR) mRNA were beyond the limit of detection.

**Table 3.** Least squares means of mRNA levels of Ca and P transporter and claudins (CLDN) as affected by dietary Ca level and microbial phytase supplementation in the colonic mucosa of growing pigs*,†,‡

Item	Dietary treatments					*P*-value		
Phytase, FTU/kg	0		500		Pooled	Ca	Phytase	Ca × Phytase
Ca, g/kg	2·0	9·6	2·0	9·6	sem			
Ca transporters								
CaBP-D9k	215	108	159	44	26·1	<0·001	<0·001	0·738
NCX1	1·77	1·56	1·72	1·74	0·139	0·350	0·521	0·258
TRPM7	20·9	20·1	20·5	20·4	0·74	0·421	0·896	0·556
TRPV5	0·034	0·025	0·022	0·011	0·008	0·030	0·004	0·778
TRPV6	1·85	1·03	1·43	0·49	0·216	<0·001	0·002	0·572
P transporters								
PiT-1	17·0	17·5	16·1	18·2	1·03	0·055	0·827	0·211
PiT-2	122	115	134	119	15·1	0·452	0·610	0·781
XPR1	9·9	10·4	10·7	10·5	0·71	0·714	0·385	0·534
Tight junctions								
CLDN-2	12·5	12·0	12·0	12·6	1·08	0·891	0·962	0·511
CLDN-4	62·5	43·8	57·6	53·8	6·82	0·027	0·603	0·136
CLDN-7	32·5^a^	29·4^b^	29·7^b^	30·3^a,b^	1·20	0·153	0·279	0·046
CLDN-10	1·90	1·19	2·17	1·24	0·264	<0·001	0·406	0·555
CLDN-12	22·8	24·4	25·2	26·0	1·50	0·246	0·072	0·711
ZO-1	5·19	5·00	5·33	5·30	0·185	0·390	0·101	0·551
Other proteins								
VDR	7·06	6·74	7·85	7·60	0·406	0·498	0·052	0·935
MINPP1	9·27	9·40	9·47	9·27	0·474	0·913	0·914	0·622
SMIT	11·9	10·8	11·8	10·7	0·65	0·026	0·893	0·957

CaBP-D9k, calbindin-D9k; IPO8, importin 8; MINPP1, multiple inositol polyphosphate phosphatase 1; NCX1, sodium-Ca exchanger; PiT-1, inorganic phosphate transporter 1; PiT-2, inorganic phosphate transporter 2; SMIT, sodium/myo-inositol cotransporter; TRPM7, transient receptor potential cation channel subfamily M member 7; TRPV5, transient receptor potential cation channel subfamily V member 5; TRPV6, transient receptor potential cation channel subfamily V member 6; VDR, vitamin D3 receptor; XPR1, xenotropic and polytropic retrovirus receptor 1; ZO-1, zonula occludens-1. Of note, transcript levels of Ca sensing receptor, calbindin-D28k, sodium-dependent phosphate transporters type IIa, IIb and IIc, CLDN-15 and alkaline phosphatase in the porcine colonic mucosa were beyond the limit of detection.

* Dietary P content was fixed at 4·7 g/kg diet.

† Data are presented as treatment means, ten replicate pens per treatment (*n* 10).

‡ Determined using absolute quantification normalised by IPO8.

^a,b^ Values lacking a common superscript within a row differ (*P* ≤ 0·05).

**Table 4.** Least squares means of mRNA levels of Ca and P transporter and claudins (CLDN) as affected by dietary Ca content and microbial phytase supplementation in the jejunal mucosa of growing pigs*,†,‡

Item	Dietary treatments					*P*-value		
Phytase, FTU/kg	0		500		Pooled	Ca	Phytase	Ca × Phytase
Ca, g/kg	2·0	9·6	2·0	9·6	sem			
Ca transporters								
CaBP-D9k	261	308	229	254	60·6	0·179	0·112	0·671
CaBP-D28k	0·099	0·064	0·071	0·060	0·028	0·333	0·486	0·605
NCX1	1·57	1·68	1·56	1·69	0·099	0·105	0·992	0·879
TRPM7	15·5	16·0	14·5	16·0	0·65	0·040	0·285	0·302
TRPV5	0·025	0·029	0·020	0·017	0·004	0·746	0·003	0·207
TRPV6	1·15	0·61	0·93	0·31	0·160	<0·001	0·003	0·630
P transporters								
NaPi-IIc	0·34	0·38	0·34	0·34	0·056	0·407	0·397	0·411
PiT-1	4·49	4·89	4·33	4·35	0·229	0·367	0·135	0·401
PiT-2	28·6	28·8	30·2	22·5	4·76	0·276	0·492	0·250
XPR1	7·90	7·17	8·10	7·20	0·480	0·024	0·734	0·797
Tight junctions								
CLDN-2	18·7	24·8	17·3	28·4	2·89	<0·001	0·598	0·233
CLDN-4	66·9	69·4	69·3	61·9	6·98	0·622	0·606	0·323
CLDN-7	23·5	23·9	24·2	24·3	1·30	0·811	0·558	0·910
CLDN-10	0·87	0·40	0·71	0·40	0·141	<0·001	0·408	0·417
CLDN-12	9·69	10·67	11·21	11·62	0·661	0·150	0·013	0·544
CLDN-15	17·1	17·4	17·8	18·5	1·60	0·665	0·433	0·851
ZO-1	3·61	3·64	3·51	3·49	0·097	0·949	0·086	0·695
Other proteins								
IAP	34·3	35·1	37·5	37·9	3·76	0·828	0·248	0·934
MINPP1	7·79	7·38	7·37	7·71	0·422	0·915	0·869	0·221
SMIT	5·69	5·60	5·21	5·51	0·412	0·719	0·339	0·511
VDR	9·1	19·5	10·1	21·1	1·52	<0·001	0·390	0·862

CaBP-D9k, calbindin-D9k; CaBP-D28k, calbindin-D28k; IAP, intestinal alkaline phosphatase; IPO8, importin 8; MINPP1, multiple inositol polyphosphate phosphatase 1; NaPi-IIc, sodium-dependent phosphate transporter type IIc; NCX1, sodium-Ca exchanger; PiT-1, inorganic phosphate transporter 1; PiT-2, inorganic phosphate transporter 2; SMIT, sodium/myo-inositol cotransporter; TRPM7, transient receptor potential cation channel subfamily M member 7; TRPV5, transient receptor potential cation channel subfamily V member 5; TRPV6, transient receptor potential cation channel subfamily V member 6; VDR, vitamin D_3_ receptor; XPR1, xenotropic and polytropic retrovirus receptor 1; ZO-1, zonula occludens-1. Of note, transcript levels of Ca sensing receptor, sodium-dependent phosphate transporter type IIa and IIb were beyond the limit of detection.

* Dietary phosphorous content was fixed at 4·7 g/kg diet.

† Data are presented as treatment means, 10 replicate pens per treatment (*n* 10).

‡ Determined using absolute quantification normalised by IPO8.

All mRNA encoding members of type II Na-Pi transporters (i.e. NaPi-IIa, NaPi-IIb and NaPi-IIc) were beyond the limit of detection in the colonic mucosa. Expression of PiT-1 tended (*P* = 0·055) to be enhanced by 8% with the intake of high Ca but not with phytase (*P* = 0·827). Transcript levels of XPR1 and PiT-2 were unaltered by the treatments.

Colonic CLDN-4 and −10 mRNA were reduced by 20 and 40%, respectively, with high Ca intake (*P* = 0·027 and <0·001, respectively), but not with phytase (*P* = 0·603 and 0·406, respectively). Phytase tended (*P* = 0·072) to enhance the expression of CLDN-12 by 8%. Furthermore, CLDN-7 mRNA was approximately 10% lower after high Ca intake only when no phytase was supplied (*P*_*interaction*_ = 0·046). In contrast, the treatments did not affect CLDN-2 and ZO-1 mRNA levels in the colon. CLDN-15, CLDN-16 and CLDN-17 mRNA in this segment were not detectable.

Phytase tended (*P* = 0·052) to enhance colonic expression of vitamin D_3_ receptor by 12% while high Ca intake had no impact (*P* = 0·498). Moreover, high dietary Ca intake reduced expression of inositol transporter by 9% (Na/myo-inositol cotransporter (SMIT), *P* = 0·026). Neither dietary Ca level nor phytase impacted the colonic expression of a candidate mucosal phosphatase (MINPP1). The other mucosal phosphatase (IAP) as well as vitamin-D_3_ 24 hydroxylase (CYP24) were not expressed.

### Jejunal expression levels

The jejunal level of CaBP-D9k mRNA was more than 4000-times higher compared with CaBP-D28k mRNA (Table 4). Dietary treatments, however, did not alter their expression levels, nor these of NCX1. Jejunal mRNA levels of TRPV6, on the contrary, were reduced (*P* < 0·003) with high Ca and phytase intake (56 and 30 % reduction, respectively). Also, TRPV5 mRNA was reduced by 32% with phytase, but not with dietary Ca intake (*P* = 0·003 and 0·746, respectively). Expression of TRPM7 was elevated by 7% with high dietary Ca (*P* = 0·040), but not with phytase intake (*P* = 0·285).

In the jejunal mucosa, expression of NaPi-IIc, PiT-1 and PiT-2 remained unaffected by the treatments. High Ca but not phytase intake decreased XPR1 mRNA (10% reduction with high Ca; *P* = 0·024 and 0·734, respectively). Noteworthy, jejunal PiT-1 and PiT-2 mRNA levels were approximately 3 times lower than those in colonic mucosa (compare Table 3). Transcripts encoding NaPi-IIa and NaPi-IIb were undetectable.

Pigs receiving high dietary Ca had approximately 50% greater (*P* < 0·001) and 50% lower (*P* < 0·001) expression of CLDN-2 and −10, respectively. Phytase inclusion elevated jejunal CLDN-12 expression by 12% (*P* = 0·013). Expression of ZO-1 tended (*P* = 0·086) to be marginally reduced with phytase. The dietary treatments did not influence the transcript levels of CLDN-4, CLDN-7 and CLDN-15.

Intake of high Ca, but not phytase, increased vitamin D_3_ receptor mRNA by 2-fold (*P* < 0·001 and 0·390, respectively). Expression of MINPP1, IAP and SMIT was not affected by the dietary treatments.

## Discussion

The accompanying data reported previously showed that Ca and P absorption in the colon were higher in growing pigs fed a phytase free compared with a phytase-supplemented diet^(2)^. We hypothesised that the phytase-induced reduction in colonic Ca and P absorption would be accompanied by a reduced expression level of Ca and P transporters in the colon. The data reported here fully supported the hypothesis: microbial phytase and high Ca intake reduced mRNA expression of TRPV5, TRPV6 and CaBP-D9k in the colonic mucosa (Table 3). A similar inhibitory effect of these two dietary factors was observed regarding the expression of TRPV6 in the porcine jejunal mucosa (Table 4). We conclude that the expression level of Ca transporters in both the jejunal (TRPV6) and colonic (TRPV5, TRPV6 and CaBP-D9k) mucosa are regulable by dietary Ca and phytase intake and that their mRNA expression level negatively correlates with the amount of absorbed Ca in the intestine.

### Effect on colonic calcium transporters, phosphatases and claudin

In line with our previous study^(2)^, post-ileal Ca absorption in pigs was also observed by others (e.g. Liu *et al.* ^(17)^), but the molecular mechanisms clarifying this observation remained to be elucidated. Here, we demonstrate that phytase inclusion tended to elevate the expression of CLDN-12 mRNA in the colonic mucosa of pigs. A previous study in mice and Caco-2 cell line demonstrated that CLDN-2 and CLDN-12 form pores permitting Ca ions to pass through, and their expression was upregulated by 1,25(OH)_2_D_3_ exposure^(18)^. This indicates that the enhanced expression of CLDN-12 in response to phytase inclusion might improve the colonic mucosal permeability to Ca.

Contrary to CLDN-12, expression of TRPV5, TRPV6 and CaBP-D9k was reduced with phytase and high Ca intake. Expression of Ca transporters in the colon of pigs has not been reported previously. *In vivo* studies in mice demonstrated that a high dietary Ca level reduced mRNA levels of TRPV6 in the proximal colon^(19)^. Using the Ussing chamber technique, the researchers in the same study further demonstrated that the application of high extracellular Ca concentration inhibited transcellular Ca absorption in proximal colon tissues of mice. Their results indicate that colonic Ca absorption, particularly active transcellular Ca absorption, is reduced by a high dietary Ca intake, which was in line with our observations in pigs. This modulation of Ca transport might be mediated via the action of PTH and vitamin D_3_^(20)^, as we observed that the inhibition of expression of TRPV5, TRPV6 and CaBP-D9k in response to high dietary Ca intake was accompanied by a lower serum PTH content. We propose that the elevated serum Ca content in response to high dietary Ca intake inhibits PTH secretion by stimulating CaSR expressed in the parathyroid gland^(21)^. A reduction of PTH, in turn, decreases circulatory 1,25(OH)_2_D_3_ to limit renal Ca reabsorption by the upregulation of renal expression of CYP24 mRNA, encoding a mitochondrial monooxygenase responsible for the modification and inactivation of bioactive 1,25(OH)_2_D_3_. The observed downregulation of TRPV5 and TRPV6 mRNA expression in the colon could be ascribed to the reduced concentration of serum 1,25(OH)_2_D_3_ since the genes of these transporters contain a vitamin D_3_ receptor -responsive element in their promoter region^(22)^. The upregulated expression of vitamin D_3_ receptor in both jejunal and colonic mucosa in response of high Ca and/or phytase intake supports this idea. In mice, Ca-sensing by CaSR located in the intestine may decrease the expression of TRPV6 and CaBP-D9k directly^(19)^. However, this mechanism seems to be unlikely in pigs since CaSR mRNA was beyond the limit of detection in both jejunal and colonic mucosa in the present study (data not shown).

We determined the mRNA expression of mucosal phosphatases (MINPP1 and IAP) and inositol transporters (SMIT) assuming that their expression level would be affected by our dietary treatments. However, IAP mRNA was beyond the limit of detection in colon and not affected by dietary treatments in jejunum. Moreover, MINPP1 mRNA was not altered by phytase inclusion or dietary Ca intake in both intestinal segments. The MINPP1 is an intercellular phosphatase located in endoplasmic reticulum. Recent *in vitro* studies in the Caco-2^(23)^ and H1299 cell line^(24)^ indicate that MINPP1 is secreted into the medium and can catalyse dephosphorylation of extracellular IP. It is, therefore, possible that MINPP1 is involved in the degradation of IP in the intestine. Previous studies in broilers indicated that high Ca intake reduced mucosal phosphatase efficacy to degrade IP in the brush-border vesicles of the small intestine^(25)^. Brun *et al.* ^(26,27)^ also reported that high Ca intake modified the activity of IAP without affecting its protein expression level in the small intestine of rats. These studies support our results and indicate that dietary Ca intake or phytase does not affect the level of IAP expression to regulate the efficacy of IAP. Our results also demonstrated that colonic expression of SMIT was significantly reduced with high dietary Ca intake although this effect was minor. We observed that high Ca intake reduced the degradation of IP in the small intestine in pigs^(2)^, which subsequently promotes the availability of IP for degradation into inositol in the colon.

The colon plays an important role in mineral and water absorption in pigs^(28)^. The reduction of mRNA expression of CLDN-4, CLDN-7 and CLDN-10 in the colon indicates that high Ca intake impaired paracellular permeability for anions and cations in the porcine colon. Unfortunately, the ion permeability of these CLDN members is still debated. Van Itallie *et al.* ^(11)^ reported that CLDN-4 could mediate Cl permeation, while Hou *et al.* ^(29)^ argued that it builds pores for Na. Experiments to unravel the ion selectivity of CLDN-7 led to controversial results; the protein may mediate transepithelial permeation of Mg^(30)^ or Cl^(31)^. Other studies (e.g. Van Itallie *et al.* ^(32)^) demonstrated the existence of two splice variants of CLDN-10, designated CLDN-10a and CLDN-10b, each with different physiological functions in mice. By contrast, Gunzel *et al.* ^(33)^ reported the existence of six different isoforms of CLDN-10 as a result of alternative splicing in mice and humans. We performed an intensive Blast search (Build Sscrofa11.1) and analysed two porcine CLDN-10 homologues: CLDN-10a (accession no. XM_021065094) and CLDN-10b (accession no. XM_021065095). In the present study, we used a primer set that hybridises to a completely identical region, shared by both isoforms in order to determine the sum expression level. Taken together, downregulation of CLDN-4, CLDN-7 and CLDN-10 may alter the mucosal paracellular permeability to traverse certain minerals across the colonic epithelial layer. It remains unknown which specific minerals are involved.

### Effect on jejunal calcium transporters and claudin

Similar to the colon, high Ca and phytase intake reduced TRPV6 expression in jejunal mucosa. Gonzalez-Vega *et al.* ^(34)^ reported that incremental dietary Ca intake linearly reduced jejunal expression of TRPV6 and CaBP-D9k, which is in agreement with our results. Noteworthy, in our study jejunal TRPV5 expression was not affected by dietary Ca level, whereas it was not reported by Gonzalez-Vega *et al.* ^(34)^. An explanation may be that TRPV5 in mice is primarily expressed in the kidney, while TRPV6 is ubiquitously expressed^(3)^. Indeed, we found that TRPV6 expression was approximately 30 and 50 times higher than TRPV5 in porcine jejunal and colonic mucosa, respectively. By contrast, renal expression of TRPV5 in pigs was approximately 6 times higher than TRPV6 (Hu *et al*. unpublished). Collectively, it appears that TRPV5 is less involved in intestinal Ca absorption compared with TRPV6. We also observed an upregulation of TRPM7 with dietary Ca intake in the jejunal mucosa. However, unlike TRPV5 and TRPV6, two channels highly selective for Ca^(35)^, TRPM7 seems to be less selective with higher affinity for Zn, Mg and Mn than Ca^(36)^. An upregulation of TRPM7 might be indicative of an antagonistic impact of a high dietary Ca against other cations.

Contrary to TRPV5 and TRPV6, CLDN-2 and CLDN-12 mRNA expression in the jejunal mucosa was enhanced by high Ca and phytase intake, respectively. This regulation pattern of CLDN-2 and CLDN-12 was in line with the soluble inorganic Ca content in the distal small intestine, which indicated that Ca was transported in the direction of the electrochemical gradient across the epithelial layer with high Ca intake but against it with low Ca intake (online Supplementary Table 1). It could be that low dietary Ca intake reduced CLDN-2 expression in order to prevent the backflow of Ca to the luminal side. By contrast, high dietary Ca intake enhanced CLDN-2 expression probably to increase the capacity of passive Ca uptake as an energy-saving process. Taken together, the inhibitory action on the expression of TRPV6, and also TRPV5 and CaBP-D9k in the colon, and stimulatory effect on Ca permeable CLDN-2 and CLDN-12 with high Ca intake suggest that paracellular Ca permeation was minor at a low but more pronounced with high Ca intake. Based on these findings, it is postulated that Ca absorption shifts from transcellular to paracellular pathway with high dietary Ca and phytase intake.

Compared with CaBP-D9k, transcript levels of CaBP-D28k were very low in the jejunal mucosa, even absent in the colonic mucosa. This indicates that CaBP-D9k may play a greater role in shuttling Ca across cytoplasm in the GIT of pigs. Christakos *et al.* ^(37)^ reviewed that CaBP-D28k was expressed highest in the avian intestine and mammalian kidney, while CaBP-D9k was observed only in mammals and was abundantly expressed in the mammalian intestine. Indeed, we observed a much higher expression of CaBP-D28k compared with CaBP-D9k in the kidney of pigs (Hu *et al*. unpublished). Thus, the distribution of CaBP-D28k and CaBP-D9k appears tissue-specific in pigs. Moreover, despite the similar names, the physiological role of CaBP-D28k may differ from CaBP-D9k. A previous *in vivo* study using transgenic mice^(38)^ indicated that mice lacking CaBP-D28k but with normal expression of CaBP-D9k developed little calcaemic abnormalities; by contrast, mice expressing only 10 % of CaBP-D9k but with normal expression level of CaBP-D28k developed hypocalcaemia and rickets. It seems that the Ca buffering function of CaBP-D28k can be largely compensated by CaBP-D9k, but not *vice versa*. It is, therefore, possible that CaBP-D9k plays a greater role in the transit of Ca across the intestinal mucosa to serve whole body Ca homeostasis.

### Effect on phosphorous transporters

Instead of NaPi-IIb, we detected NaPi-IIc in the porcine jejunum. Moreover, all type II transporters were absent in the colonic mucosa. Apparently, NaPi-IIc is the most important Na-P cotransporter in the small intestine of pigs. Wubuli *et al.* ^(39)^ investigated the tissue-wide mRNA expression status of all currently annotated P transporters. Their results combined with our observation indicate that NaPi-IIc is the only type II P transporter abundantly expressed in the GIT of pigs and that this isotype contributes to intestinal P absorption. We demonstrated that high Ca uptake reduced intestinal P absorption in the small intestine of these pigs^(2)^. Here, we found that the level of NaPi-IIc mRNA was neither affected by dietary Ca level nor phytase inclusion, suggesting that the reduction of P absorption was attributed to a lower NaPi-IIc activity which was regulated at the posttranscription level, e.g. vesicle trafficking of NaPi-IIc to-and-from the apical membrane.

We observed a nominally (*P* = 0·055) elevated expression of PiT-1 mRNA in the colon when pigs were fed a high Ca compared with low Ca diet. Previous transgenic studies showed that overexpression of PiT-1 enhanced serum P and reduced serum Ca in both rats^(40)^ and mice^(41)^. These data raise the possibility that high dietary Ca led to insoluble Ca–P complexes impairing intestinal P absorption and serum P content. A higher expression of PiT-1 may increase the capacity of P absorption in the pig’s colon to support systemic P homeostasis.

Paracellular P permeation might also substantially contribute to intestinal P absorption in pigs. Soluble inorganic P content was much lower in the serum than in the digesta in all GIT segments (online Supplementary Table 1), which generated a wide electrochemical gradient difference of P across the epithelium cell. To the knowledge of the authors, paracellular P absorption in pigs has not been reported before. Using the *in situ* ligated intestinal loop technique, Marks *et al.* ^(42)^ demonstrated in rats that only 30 % of the intestinal P absorption was Na dependent, irrespective of P content in the buffer solution instilled into the GIT segments. Their results suggest that P absorption might be primarily mediated via paracellular permeation in the GIT. This assumption is in line with an *in vivo* study in pigs^(43)^ which demonstrated that incremental dietary P level linearly enhanced total tract P digestibility. Thus, P absorption is highly dependent on luminal P content, which suggests that paracellular P absorption may be substantial in pigs. However, the mechanism for P permeation remains unknown.

In conclusion, our data suggested that Ca absorption shifts from transcellular to paracellular pathways when dietary Ca is increased from deficient to abundant and also when Ca availability is enhanced with microbial phytase supplementation, as evidenced by downregulation of jejunal and colonic genes related to transcellular Ca absorption and upregulation of Ca pore-forming claudins (CLDN-2 and CLDN-12). Paracellular P absorption may be substantial in pigs as the expression of genes related to transcellular P absorption remain mostly unaffected by dietary Ca and phytase content.
